# Global ocean synoptic thermocline gradient, isothermal-layer depth, and other upper ocean parameters

**DOI:** 10.1038/s41597-019-0125-3

**Published:** 2019-07-10

**Authors:** Peter C. Chu, Chenwu Fan

**Affiliations:** Naval Ocean Analysis and Prediction Laboratory, Department of Oceanography, Naval Postgraduate School, Monterey, CA 93943 USA

**Keywords:** Climate change, Physical oceanography

## Abstract

Different from the existing global ocean climatological datasets of isothermal layer (ITL) depth (*h*), a global ocean synoptic dataset of *h* along with other parameters has been established from temperature profiles of the National Centers for Environmental Information (NCEI) world ocean database 1961–2017. The exponential leap-forward gradient method was used to identify *h* and thermocline gradient (*G*) from each temperature profile measured by expendable bathythermograph (XBT) and conductivity, temperature, depth (CTD) instruments. Due to quality and vertical resolution of the profiling data, the numbers of (*G*, *h*) pairs are 446,811 out of 964,942 CTD profiles and 755,086 out of 2,303,433 XBT profiles. With the given *h*, other parameters such as ITL heat content (*H*_*ITL*_), sea surface temperature (*SST*), temperature below ITL (*T*_*m*_), and quality measures (*Q*, *I*) indices are provided. Altogether, the dataset contains 1,201,897 temporally and horizontally varying sets of (*G*, *h*, *H*_*ITL*_, SST, *T*_*m*_, *Q*-index, *I-*index). Note that we added 200° to the longitude. This synoptic dataset is located on the NCEI website for public use.

## Abstract (summary)

**Table:** 

Design Type(s)	source-based data analysis objective
Measurement Type(s)	marine pelagic feature
Technology Type(s)	digital curation
Factor Type(s)	
Sample Characteristic(s)	Earth (Planet) • ocean biome

Machine-accessible metadata file describing the reported data (ISA-Tab format)

## Background & Summary

Existing global datasets of isothermal layer (ITL) depth (*h*) are all climatologies on regular grids with 1° horizontal resolution and standard World Ocean Atlas (WOA) vertical depths, either computed from 3D gridded temperature climatology^1,2^ or identified from observational temperature profiles and then averaged in 1° or 2° cells^3,4^. Such datasets provide climatological monthly mean gridded values. However, many processes occurring in the ITL cause *h* to change on diurnal, seasonal, to interannual time scales^5–7^, which cannot be represented by the existing climatological monthly mean datasets.

Besides, underneath the ITL, there exists a thermocline with a strong gradient (*G*). Both *h* and *G* are important parameters in studying climate variability, interaction between atmosphere and oceans, and ocean prediction^8^. For example, the thermocline affects the heat exchange between the ITL and a deeper layer, and changes the ITL heat content (*H*_*ITL*_), influences the evolution of the sea surface temperature (SST), and in turn the heat/moisture fluxes and upward long wave radiation at the surface^9^. Thus, establishment of a synoptic dataset of (*h*, *G*, *H*_*ITL*_, SST) from temperature profiles becomes urgent.

The traditional approach to determine *h* from temperature profiles is because of a single *near-zero* gradient in the ITL using difference and gradient methods. The difference method requires the deviation of temperature from its value at a reference depth (*z*_*ref*_) to be smaller than a certain fixed value, which varies among 1.0 °C^10^, 0.8 °C^11,12^, 0.2 °C^4,13,14^, to 0.1 °C^15^. The reference level changes from near the surface^12^ to 10 m depth^4,15^. The gradient method requires the vertical temperature gradient ∂*T*/∂*z* to be smaller than a certain fixed value, which varies from 0.015 °C/m^16^ to 0.025 °C/m^17,18^. A recent approach is to use the transition from the *near-zero* gradient in the ITL to the *non-zero* gradient in the thermocline to determine *h*. This leads to a maximum curvature method^19–22^. Large errors may occur in noisy profile data since the curvature involves the calculation of second derivative versus depth^20,22^. To improve the curvature method, the optimal linear fitting^23^ and maximum angle^24^ methods were developed with capability of handling noisy profile data. However, these two methods are iterative and not as straightforward as earlier methods such as the difference, gradient, and maximum curvature methods. Furthermore, none of these methods determines *G*.

Recently, the exponential leap-forward gradient (ELG)^25^ was developed to archive (*G*, *h*, SST) and temperature below the ITL (*T*_*m*_) simultaneously from a temperature profile, and has been verified as optimal among all the existing methods with the highest skill score using the *Q*-index^22^ and the lowest Shannon information entropy (representing the least uncertainty)^25^. Vertical integration of temperature profile from the surface (z = 0) down to the base of the ITL (z = −h) leads to the ITL heat content (*H*_*ITL*_). The benefit of using *H*_*ITL*_ rather than heat content within fixed depths such as *H*_700_ for the upper 700 m is that *H*_*ITL*_ is fully determined by the upper ocean mixed layer dynamics and directly interacts with the atmosphere but *H*_700_ is not. Dynamics is more complicated for *H*_700_ than for *H*_*ITL*_.

We analysed the global ocean CTD and XBT temperature profiles (1961–2017), downloaded from the NCEI website https://www.nodc.noaa.gov/OC5/WOD/pr_wod.html in May 2018, with the ELG method. After that, the (*G*, *h*) data pairs were generated by the ELG method from 446,811 out of 964,942 CTD profiles and 755,086 out of 2,303,433 XBT profiles. The lower number of (*G*, *h*) data pairs compared to the observational temperature profiles is due to their quality and vertical resolution. Nine criteria are used for the data filtering such as too few data points between 10 m and 40 m, too few total observational points, maximum depth less than 20 m and so on. Please see MATLAB function (getgradient.m). The NCEI/WOD observational XBT profiles (no correction) were used because the attached algorithm is for analysing observational temperature profiles with real vertical data distribution (not on the standard depths). The quality of the (*G*, *h*) data were identified by both *Q*-index^22^ and identification index (see the Technical Verification Section). Altogether, the dataset contains 1,201,897 sets of (*G*, *h*, *H*_*ITL*_, *SST*, *T*_*m*_, *Q*-index, *I*-index), and is located at the NCEI website for public use.

## Methods

### ELG Method

**Fig. 1:**
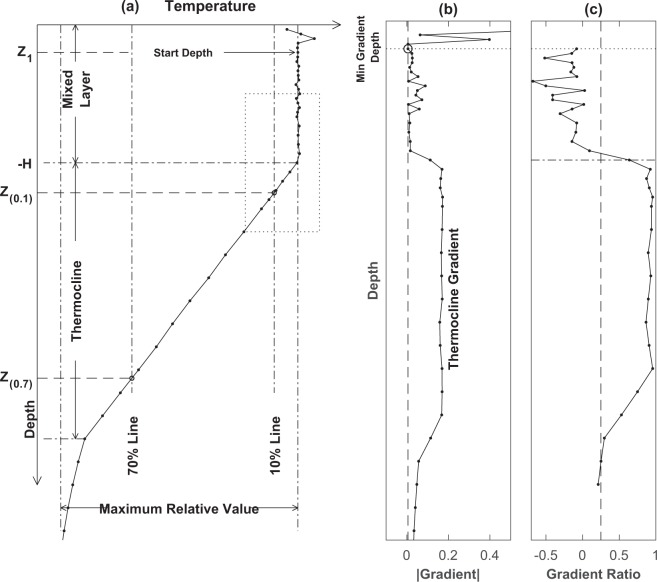
Characteristics of the isothermal layer and thermocline. (**a**) Temperature profile with the illustrated z_(0.1)_ and *z*_(0.7)_. (**b**) Vertical gradient and the defined *z*_1_. (**c**) Gradient ratio.

The ELG method^25^ was used to process observational temperature profile data to obtain (*G*, *h*). It contains four steps: (1) estimating the ITL gradient (near-zero), (2) identifying the thermocline gradient *G*, (3) to computing the vertical gradient at each depth (non-dimensionalized by *G*), and (4) to determining *h* with a given threshold (or user input) to separate the near-zero gradient layer (i.e., the ITL) and the non-zero gradient layer (i.e., the thermocline). Figure 1 illustrates the procedures of this method.

#### Step 1

Temperature profile data are often noisy, sometimes with unrealistically high vertical gradients near the surface. The reference levels (*z*_*ref*_) of (0 m, −3 m, −10 m) are used for the difference method criteria in determination of *h* to reduce such noise. Thus, a layer with depth deeper than *z*_*ref*,_ say 20 m, represents the vertical temperature variation of the upper layer. A depth (*z*_1_) with a minimum gradient

1 $${\left|\frac{{\rm{\Delta }}T}{{\rm{\Delta }}Z}\right|}_{z={z}_{1}}=\mathop{Z\ge -20\,{\rm{m}}}\limits^{min}\left|\frac{{\rm{\Delta }}T}{{\rm{\Delta }}z}\right|,$$

is identified within this layer (i.e., 0 ≥ *z* ≥ −20 m). This minimum gradient at *z*_1_ is the best representation of the ITL gradient (Fig. 1a).

#### Step-2

Let an observational temperature profile starting from *z*_1_ to any depth *z*_*k*_ be represented by [*T*(*z*_*k*_), *k* = 1, 2, …, *b*] with *z*_*b*_ the bottom of the profile. The vertical temperature difference from *z*_1_ to *z*_*b*_, *T*_*d*_ = *T*(*z*_1_) − *T(z*_*b*_), is the variation of temperature across the ITL, thermocline, and deep layer. Since the vertical gradient is strongest in the thermocline and weakest in the ITL (Fig. 1b,c), it is reasonable to assume that the vertical temperature difference inside the ITL is within 10% of *T*_*d*_. Since the deep layer is usually not vertically well mixed, the temperature variation is more in the deep layer than in the ITL. Thus, the main part of the thermocline can be roughly identified between the upper depth [z_(0.1)_] with 10% temperature difference to z_1_ comparing to *T*_*d*_

2a $$T({z}_{1})-T({z}_{(0.1)})=0.1{T}_{d},$$

and the lower depth [*z*_(0.7)_] with 70% temperature difference to z_1_ comparing to *T*_*d*_ (Fig. 1a).

2b $$T({z}_{1})-T({z}_{(0.7)})=0.7{T}_{d}.$$

Let (*N*_*th*_ + 1) be the number of vertical data points for the closed interval [z_(0.1)_, *z*_(0.7)_] with [*T*_0_ = *T*(*z*_(0.1)_), *T*_*Nth*_ = *T*(*z*_(0.7)_)], and (*T*_*i*_, *i* = 0, 1, 2, …, *N*_*th*_ − 1) in between. The ordinary vertical gradients are calculated from the depth *z*_(0.1)_ to each depth through *z*_(0.7)_,

3 $${G}_{i}=\left[T({z}_{(0.1)})-{T}_{i}\right]/[{z}_{(0.1)}-z{| }_{{T}_{i}}],\quad i=1,2,\,\ldots ,{N}_{th}.$$

Here, the gradient data [*G*_1_, *G*_2_, …, *G*_*Nth*_] are not normally distributed. Thus, the overall feature [i.e., *thermocline gradient* (*G*)] is represented by the median,

4 $$G={\rm{Median}}\left[{G}_{1},{G}_{2},\,\ldots ,{G}_{{N}_{th}}\right].$$

If the computed *G* is extremely small,

5 $$G\le 0.001\,^\circ {\rm{C}}\,{{\rm{m}}}^{-1}$$

the thermocline vanishes. Since the temperature difference between *z*_(0.7)_ and *z*_*b*_ is only 30% of *T*_*d*_, and the vertical distance of neighbouring data points increases with depth below z_(0.7)_, the gradient below z_(0.7)_ is also extremely small. The ITL extends to the bottom of the profile *z*_*b*_.

#### Step 3

Let *N*_*g*_ be the number of the vertical data points between *z*_1_ and *z*_(0.7)_ (inclusive), and let $N=\left\langle {{\rm{log}}}_{2}({N}_{g})\right\rangle$ with the bracket indicating the integer part of the real number inside. *N* is much smaller than *N*_*g*_. Starting from *z*_1_, the (*N* + 1) exponential leap-forward gradients (ELGs) are calculated at each depth *z*_*k*_ [between *z*_1_ and *z*_(0.7)_]

6 $${D}_{n}T({Z}_{k})=[T({Z}_{k})-T({T}_{k+{2}^{n}})]/[{Z}_{k}-{Z}_{k+{2}^{n}}],\,n=1,\,2,\,\ldots ,\,N.$$

where the computation stops if ${z}_{k+{2}^{n}}$ is equal to or deeper than *z*_*b*_. It is also noted that *D*_*n*_ is an identifier and not a numerical value. The averaged value among (*N* + 1) gradients [*D*_0_*T*(*z*_*k*_), *D*_1_*T*(*z*_*k*_), …, *D*_*N*_*T*(*z*_*k*_)] is computed by

7 $${G}_{\ast }({z}_{k})=\left[\sum _{n=1}^{N}{D}_{n}T({z}_{k})\right]/N,$$

which represents the gradient effectively at the depth *z*_*k*_ with capability to filter out noises in the gradient calculation.

#### Step 4

Since ${G}_{\ast }({z}_{k})\approx 0$ if *z*_*k*_ in the ITL; ${G}_{\ast }({z}_{k})=G$ if *z*_*k*_ in the thermocline,

8 $$R({z}_{k})\equiv {G}_{\ast }({z}_{k})/G=\left\{\begin{array}{l} \sim 0,\quad {z}_{k}\,{\rm{in}}\,{\rm{the}}\,{\rm{ITL}}\\  \sim 1,\quad {z}_{k}\,{\rm{in}}\,{\rm{the}}\,{\rm{thermocline}}\end{array}\right.$$

A threshold of the gradient ratio, *R*(*z*_*k*_), is needed to determine if *z*_*k*_ is in the ITL or the thermocline. We make it a user input parameter. Since there is no layer between the ITL and thermocline, and gradient in the thermocline is near 1, this threshold should be reasonably near 1 to be included in the thermocline. Here, 0.8 is suggested, but readers can change it in their practice.

### Data filtering

The ELG method requires a certain quality of the profile data. A temperature profile is flagged if it has the following features: (1) number of data points ≤ 2 between 10 and 40 m depths, (2) total number of data points ≤ 5, (3) maximum depth < 20 m (not reaching thermocline), (4) upmost data point deeper than 50 m (no way to identify the ITL), (5) variance above 20 m > variance below 20 m, (6) range (*T*_max_ − *T*_min_) < 1.0 °C, (7) too small thermocline gradient (<0.001 °C/m) (no thermocline), (8) big change (>5 °C) between two neighboring data points. Finally, 446,811 out of 964,942 CTD profiles and 755,086 out of 2,303,433 XBT profiles are not flagged (i.e., normal). Thus, 1,201,897 sets of (*G*, *h*, *H*_*ITL*_, *SST*, *T*_*m*_, *Q*-index, *I*-index) have been established. Note that SST here is the temperature at the ITL base (z = −*h*). The bulk SST is easily obtained from *H*_*ITL*_ and *h* or from the first depth level of the profile.

### Data Traced Back to the WOD Profiles

The dataset contains 1,201,897 sets of (*G*, *h*, *H*_*ITL*_, *SST*, *T*_*m*_, *Q*-index, *I*-index). Each set has exactly the same metadata as the original temperature profile in the WOD such as time, location (longitude, latitude), country code, WOD cruise identifier, and WOD unique cast. Note that we added 200° to the WOD latitudes. With these parameters, each set of (*G*, *h*, *H*_*ITL*_, *SST*, *T*_*m*_, *Q*-index, *I-*index) is easy to trace back to the original WOD temperature profile.

### Use of WOD CTD (2013-14) for Illustration

The WOD dataset (1961–2017) contains 964,942 CTD and 2,303,433 XBT profiles with one file for one profile. We uploaded the WOD CTD temperature data (2013–2014) with 55,554 profiles in the folder named ‘wod20132014CTD’ and the derived dataset of (*G*, *h*, *H*_*ITL*_, *SST*, *T*_*m*_, *Q*-index, *I-index*) named ‘WODCTD1314ELGtemp.nc’ to the Naval Postgraduate School website http://faculty.nps.edu/pcchu/ocean_data.htm (Item 12) for readers to practise. The following MATLAB codes are ready to use for this sub-dataset. It is easy to change dataset name to process other profile data.

## Data Records

This global dataset for ocean synoptic thermocline gradient, isothermal-layer depth, and other upper ocean parameters^26^ is publicly available at the NOAA/NCEI data repository as a NetCDF file, which includes data citation, dataset identifiers, metadata, and ordering instructions. The dataset is located at the NCEI website (10.25921/dgak-7a43) for public use.

## Technical Validation

**Fig. 2:**
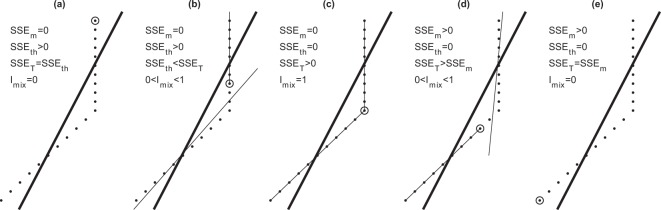
The identification index to represent the quality of the ITL depth determination. (**a**) ITL not existence or ITL in existence, but not identified (*h* = 0). (**b**) Identified ITL depth shorter than the real ITL depth. (**c**) Perfectly identified ITL depth. (**d**) Identified ITL depth longer than the real ITL depth. (**e**) Identified ITL depth at *z*_(0.7)_, i.e., thermocline not existence. Here, the ITL depth is marked by a circle. The thick line in each figure is a single linear function fitted to the temperature profile data.

The key parameter of this dataset is *h*. The error at the data point is defined by the difference between the fitted and observed temperatures. The whole temperature profile data fitted to a single linear function (thick lines in Fig. 2) represents the maximum error since it disregards the existence of ITL and thermocline. Such a maximum error is represented by the total sum of the square error (SSE_T_). After the ITL depth is identified, two lines are used to fit the observational data to get the fitted profile with the first one (near zero gradient in the ITL) from the top to the ITL base (circles in Fig. 2) and the second one (non-zero gradient in the thermocline) from the ITL base to the bottom of the profile. Such fitting has the errors in the ITL represented by the sum of the square error in ITL (SSE_ITL_) and in the thermocline represented by the sum of the square error in thermocline (SSE_TH_). The identification index (called *I*-index) is defined by

9 $${I}_{ITL}=\sqrt{1-(SS{E}_{ITL}+SS{E}_{TH})/SS{E}_{T}}.$$

**Fig. 3:**
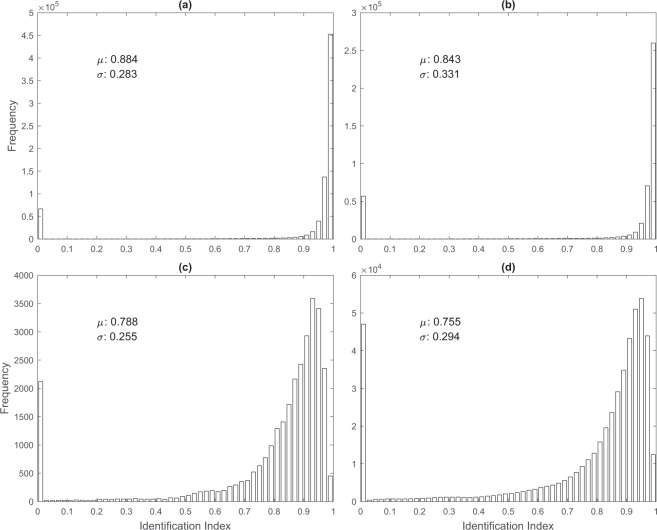
Histograms of the identification index showing the quality of determination of the ITL depth. (**a**) WOD/XBT data. (**b**) WOD/CTD data. (**c**) WOA annual 1° × 1°. (**d**) WOA annual 0.25° × 0.25°.

If an ITL exists, but is not identified (*h* = 0) (Fig. 2a), SSE_ITL_ = 0, SSE_TH_ = SSE_T_; which gives *I*_*ITL*_ = 0. If the identified *h* is shorter than the real one (Fig. 2b), SSE_ITL_ = 0, SSE_TH_ > 0, SSE_TH_ < SSE_T_; which leads to 0 < *I*_*ITL*_ < 1. If the identified *h* is the same as the real one (Fig. 2c), SSE_ITL_ = 0, SSE_TH_ = 0, SSE_T_ > 0; which makes *I*_*ITL*_ = 1. If the identified *h* is longer than the real one (Fig. 2d), SSE_ITL_ > 0, SSE_TH_ = 0, SSE_TH_ < SSE_T_; which leads to 0 < *I*_*ITL*_ < 1. If the identified *h* reaches the bottom of the thermocline [i.e., at z_(0.7)_] (Fig. 2e), SSE_TH_ = 0, SSE_ITL_ = SSE_T_; which gives *I*_*ITL*_ = 0. The histograms of *I*_*ITL*_ show high quality of the ELG method for identifying ITL depth using the WOD/XBT data with the mean *I*_*ITL*_ of 0.884 (Fig. 3a), the WOD/CTD data with the mean *I*_*ITL*_ of 0.843 (Fig. 3b). However, the quality is lower with the climatology dataset such as World Ocean Atlas (WOA) 2013, downloaded from the website: https://www.nodc.noaa.gov/OC5/woa13/woa13data.html. The mean *I*_*ITL*_ is 0.788 using the WOA-13 on 1° resolution (WOA13-1°) (Fig. 3c) and 0.755 using the WOA-13 on 0.25° resolution (WOA13-0.25°) (Fig. 3d). The low scores may be caused by the coarser vertical resolution in WOA dataset than in the WOD/CTD and WOD/XBT. Besides, the ELG method has the highest score of the commonly used Q-index^22,25^. Quite a few zero values for *I*_*ITL*_ in Fig. 3 indicate that no ITL or thermocline can be identified from those profiles.

**Fig. 4:**
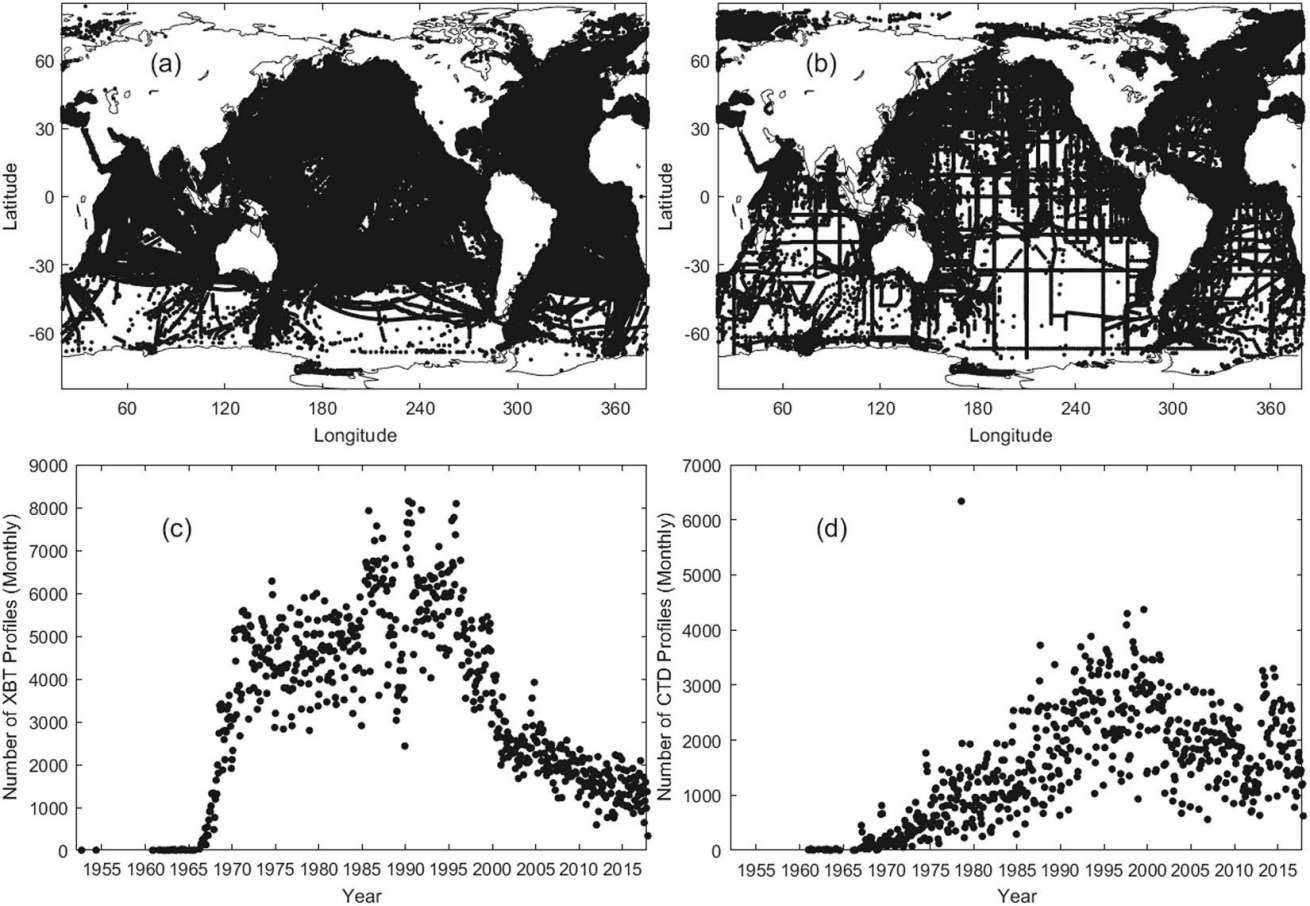
Temperature profiles were processed by the ELG method. (**a**) Horizontal distribution of 755,086 WOD/XBT stations. (**b**) Horizontal distribution of 446,811 WOD/CTD stations. (**c**) Monthly number of the WOD/XBT profiles. (**d**) Monthly number of the WOD/CTD profiles.

Figure 4 shows the spatial and temporal distributions of the derived 446,811 sets (from CTD) and 755,086 sets (from XBT) of (*G*, *h*, *H*_*ITL*_, *SST*, *T*_*m*_, *Q*-index, *I*-index). The longitude range of this dataset has the same range as the WOD from −180° to 180°. The values of the horizontal axis in Fig. 4a,b had 200° added for the sake of plotting. The dataset covers the global oceans pretty well except the Southern Ocean, where there are data-poor regions. The WOD XBT (Fig. 4c) and CTD (Fig. 4d) temperature profile data increase drastically after 1970.

**Fig. 5:**
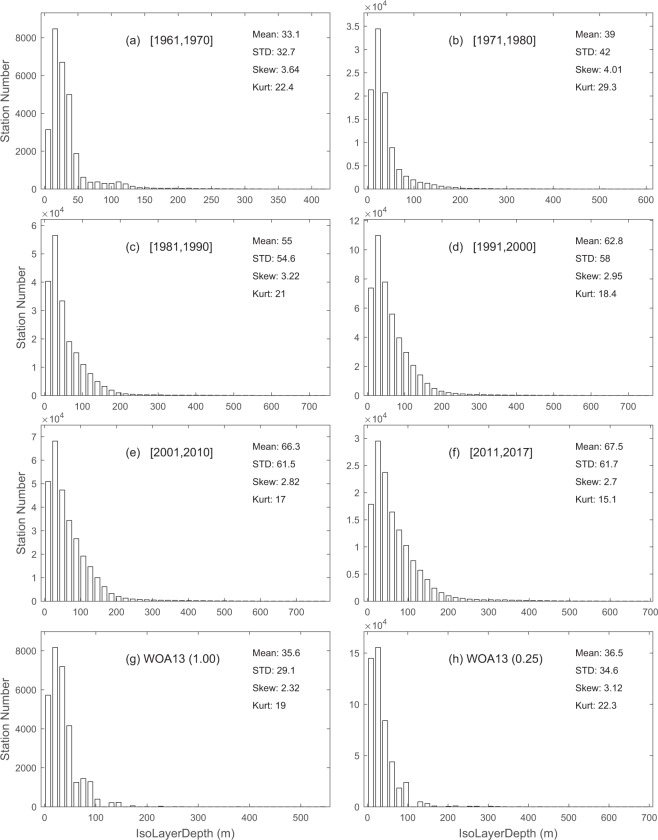
Histograms showing decadal variation for the isothermal layer depth (*h*) in comparison to the climatology. (**a**) 1961–1970. (**b**) 1971–1980. (**c**) 1981–1990. (**d**) 1991–2000. (**e**) 2001–2010. (**f**) 2011–2017. (**g**) WOA annual 1° × 1°. (**h**) WOA annual 0.25° × 0.25°.

**Fig. 6:**
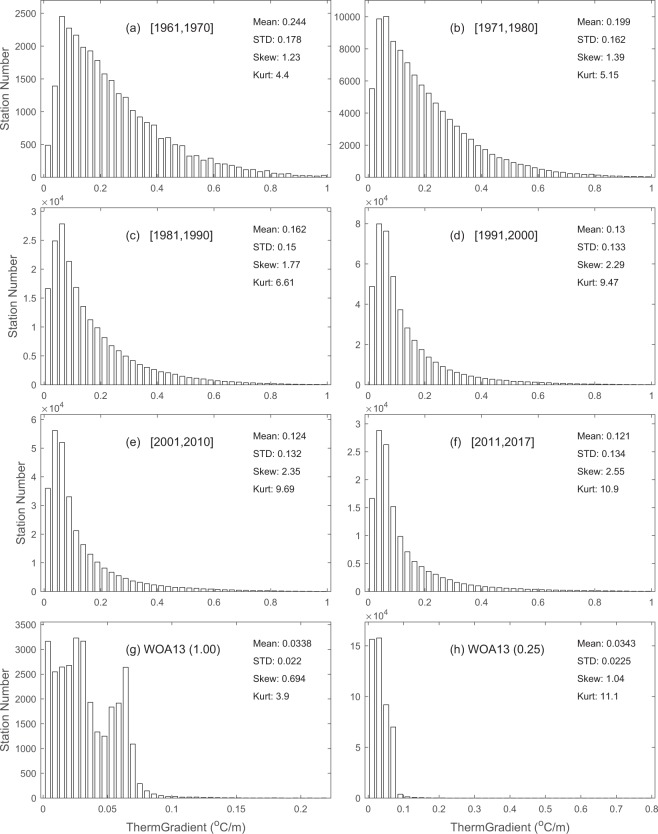
Histograms showing decadal variation for the thermocline gradient (*G*) in comparison to the climatology. (**a**) 1961–1970. (**b**) 1971–1980. (**c**) 1981–1990. (**d**) 1991–2000. (**e**) 2001–2010. (**f**) 2011–2017. (**g**) WOA annual 1° × 1°. (**h**) WOA annual 0.25° × 0.25°.

**Fig. 7:**
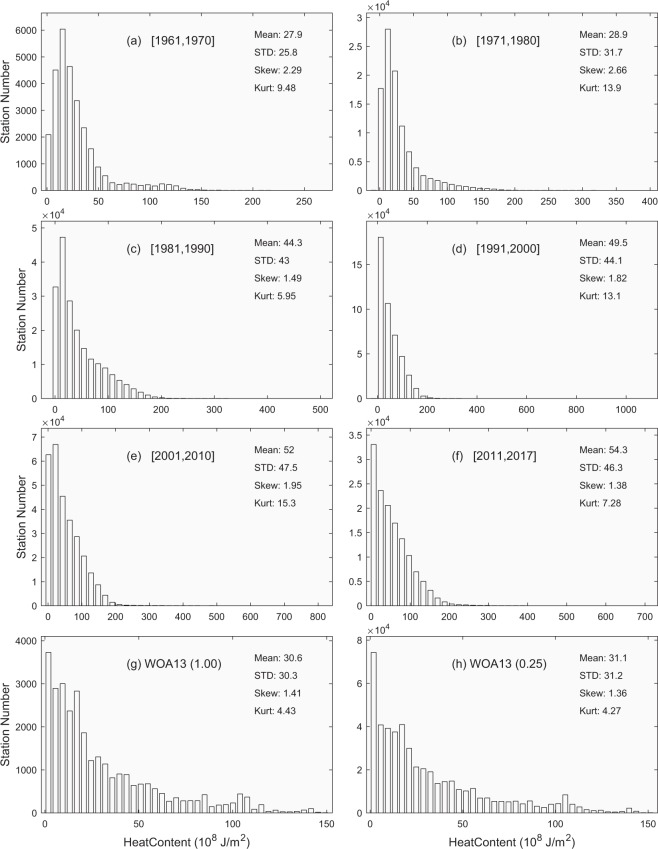
Histograms showing decadal variation for the isothermal layer heat content (*H*_*ITL*_) in comparison to the climatology. (**a**) 1961–1970. (**b**) 1971–1980. (**c**) 1981–1990. (**d**) 1991–2000. (**e**) 2001–2010. (**f**) 2011–2017. (**g**) WOA annual 1° × 1°. (**h**) WOA annual 0.25° × 0.25°.

Differently from the existing climatological datasets of *h*, this dataset provides variabilities on various time scales. Here, we take decadal variability as an example for illustration. To do so, we divided the time from 1961 to 2017 into six periods: 1961–1970, 1971–1980, 1981–1990, 1991–2000, 2001–2010, and 2011–2017, along with the climatology WOA-1° and WOA-0.25° for comparison. For each period, we used the global data to construct the histograms of *h* (Fig. 5), *G* (Fig. 6), and *H*_*ITL*_ (Fig. 7). They clearly show temporally varying (on decadal time scales) non-Gaussian distributions with strong positive skewness and higher values (>3) of kurtosis. Furthermore, we calculated the statistical parameters for variables (*h*, *G*, *H*_*ITL*_) within each period to determine the decadal variabilities.

Table 1 shows the decadal variations of statistical parameters for the global *h* with a monotonically increasing mean (standard deviation) from 33.1 m (32.7 m) during 1961–1970 to 67.5 m (61.7 m) during 2011–2017, and monotonically decreasing skewness (kurtosis) from 4.01 (29.3) during 1971–1980 to 2.70 (15.1) during 2011–2017. There is no clear pattern for skewness and kurtosis for *h*. Table 2 shows the decadal variations of statistical parameters for the global *G* with a monotonically decreasing mean (standard deviation) from 0.244 °C/m (0.178 °C/m) during 1961–1970 to 0.121 °C/m (0.134 °C/m) during 2011–2017, and monotonically increasing skewness (kurtosis) from 1.23 (4.40) during 1961–1970 to 2.55 (10.9) during 2011–2017. Table 3 shows the decadal variations of statistical parameters for the global *H*_*ITL*_ with a monotonically increasing mean (standard deviation) from 2.79 × 10^9^ J/m^2^ (2.58 × 10^9^ J/m^2^) during 1961–1970 to 5.43 × 10^9^ J/m^2^ (4.63 × 10^9^ J/m^2^) during 2011–2017. All the statistical parameters are comparable between the synoptic and climatological datasets except the mean and standard deviation of *G*, which were much lower in the climatology datasets such as (0.034 °C/m, 0.022 °C/m) in the WOA13-1°, and (0.034 °C/m, 0.023 °C/m) in the WOA13-0.25°.

**Table 1:** Decadal variation of statistical characteristics of the global isothermal layer depth (*h*) in comparison to climatology.

	Mean (m)	Standard Deviation (m)	Skewness	Kurtosis
1961–1970	33.1	32.7	3.64	22.4
1971–1980	39.0	42.0	4.01	29.3
1981–1990	55.0	54.6	3.22	21.0
1991–2000	62.8	58.0	2.95	18.4
2001–2010	66.3	61.5	2.82	17.0
2011–2017	67.5	61.7	2.70	15.1
WOA (1°)	35.6	29.1	2.32	19.0
WOA (0.25°)	36.5	34.6	3.12	22.3

**Table 2:** Decadal variation of statistical characteristics of the global thermocline gradient (*G*) in comparison to climatology.

	Mean (°C/m)	Standard Deviation (°C/m)	Skewness	Kurtosis
1961–1970	0.244	0.178	1.23	4.40
1971–1980	0.199	0.162	1.39	5.15
1981–1990	0.162	0.150	1.77	6.61
1991–2000	0.130	0.133	2.29	9.47
2001–2010	0.124	0.132	2.35	9.69
2011–2017	0.121	0.134	2.55	10.90
WOA (1°)	0.034	0.022	0.89	3.90
WOA (0.25°)	0.034	0.023	1.04	11.10

**Table 3:** Decadal variation of statistical characteristics of the global isothermal layer heat content (*H*_*ITL*_) in comparison to climatology.

	Mean (10^8^J/m^2^)	Standard Deviation (10^8^J/m^2^)	Skewness	Kurtosis
1961–1970	27.9	25.8	2.29	9.48
1971–1980	28.9	31.7	2.66	13.90
1981–1990	44.3	43.0	1.49	5.95
1991–2000	49.5	44.1	1.82	13.10
2001–2010	52.0	47.5	1.95	15.30
2011–2017	54.3	46.3	1.38	7.28
WOA (1°)	30.6	30.3	1.41	4.43
WOA (0.25°)	31.1	31.2	1.36	4.27

## Supplementary Information

### ISA-Tab metadata file

Download metadata file

### Supplementary information

Supplementary Material

## Data Availability

We present custom codes with the MATLAB to determine (*G*, *h*, *I*_*ITL*_) from an individual temperature profile (see ‘Supplementary Material’). The main program is ‘ThermoclineMLD.m’, which contains four Matlab functions: ‘validata.m’ to filter out bad profiles, ‘getgradient.m’ to calculate the vertical gradients between z_(0.1)_ and z_(0.7)_, ‘ELGCore.m’ to calculate the ELGs from z_1_ to z_(0.7)_, and “Iindex.m’ to calculate the Identification index (*I*_*ITL*_) for the technical validation (see the Technical Validation Section). The code ‘getgradient.m’ can use temperature or potential density profiles to obtain the ITL and thermocline gradient or mixed layer and pycnocline gradient, but only the work with temperature profiles will be explained in this data descriptor. Interested readers may use our MATLAB codes to analyse the WOD 2013–14 CTD temperature profiles and to get the derived dataset (*G*, *h*, *SST*, *T*_*m*_, *H*_*ITL*_, *Q*-Index, *I*-index).
