# Supplementary material for: Global ocean synoptic thermocline gradient, isothermal-layer depth, and other upper ocean parameters
Source: Sci Data. 2019 Jul 10;6:119. doi: 10.1038/s41597-019-0125-3 (PMC6620299; doi:10.1038/s41597-019-0125-3)
Supplement: Supplementary file 2 — Supplementary Material [file 41597_2019_125_MOESM2_ESM.docx]

### Supplementary Material

***MATLAB main program (ThermoclineMLD.m)***

% main program 'ThermoclineMLD.m' to analyze NCEI/WOD CTD (2013-2014) profiles

% Input the characteristic sea-water density, refD=1025 kg m^-3^

% Input the sea-water specific heat capacity, Cp=3985 J kg^-1^K^-1^

refD=1025; Cp=3985;

oldFormat = netcdf.setDefaultFormat('NC_FORMAT_64BIT');

% warning off MATLAB:xlswrite:AddSheet;

% Build up the NetCDF interface using the Matlab (Matlab v.2008 and up)

woddir='wod20132014CTD/';

nccid=netcdf.create('WODCTD1314ELGtemp.nc','clobber');

netcdf.putAtt(nccid,-1,'Description',['Isothermal Layer Depth using the ELG(OMES) ',...

'Method for the WOD CTD Observational Dataset']);

% Set up dimension for the netcdf files (dim8, dim13, dim17, and dimprf) with dimprf for profiles.

dim8=netcdf.defDim(nccid,'Dim8',8);

dim17=netcdf.defDim(nccid,'Dim17',17);

dim13=netcdf.defDim(nccid,'Dim13',13);

dimprf=netcdf.defDim(nccid,'Nprof',0);

flid=netcdf.defVar(nccid,'netcdffile','char',[dim17,dimprf]);

netcdf.putAtt(nccid,flid,'Description','Original NetCDF profile name');

Cotryid=netcdf.defVar(nccid,'Country','char',[dim13,dimprf]);

Cruisid=netcdf.defVar(nccid,'WOD_cruise_identifier','char',[dim8,dimprf]);

netcdf.putAtt(nccid,Cruisid,'comment','two byte country code + WOD cruise number (unique to country code)');

prfid=netcdf.defVar(nccid,'wod-unique_cast','long',dimprf);

netcdf.putAtt(nccid,prfid,'cf_role','profile_id');

timeid=netcdf.defVar(nccid,'time','double',dimprf);

netcdf.putAtt(nccid,timeid,'unit', 'days since 1770-01-01 00:00:00');

dateid=netcdf.defVar(nccid,'date','long',dimprf);

netcdf.putAtt(nccid,dateid,'comment','YYYYMMDD');

latid=netcdf.defVar(nccid,'Latitude','float',dimprf);

lonid=netcdf.defVar(nccid,'Longitude','float',dimprf);

TGrdid=netcdf.defVar(nccid,'ThermGrd','float',dimprf);

netcdf.putAtt(nccid,TGrdid,'Description','Thermocline Gradient (dT/dz)');

netcdf.putAtt(nccid,TGrdid,'unit','oC/m');

mxdid=netcdf.defVar(nccid,'IsoLayerDepth','float',dimprf);

netcdf.putAtt(nccid,mxdid,'Description','Isothermal Layer Depth');

netcdf.putAtt(nccid,mxdid,'unit','m');

MTid=netcdf.defVar(nccid,'SST','float',dimprf);

netcdf.putAtt(nccid,MTid,'Description','Isothermal Layer Temperature');

netcdf.putAtt(nccid,MTid,'units','oC');

HCid=netcdf.defVar(nccid,'HeatContent','float',dimprf);

netcdf.putAtt(nccid,HCid,'Description','Isothermal Layer Heat Content with the reference density 1025kg/m^3');

netcdf.putAtt(nccid,HCid,'unit','J/m^2');

Iiid=netcdf.defVar(nccid,'Ii_MixedLayerDepth','float',dimprf);

netcdf.putAtt(nccid,Iiid,'description','Mixed Layer Depth Identification Index');

netcdf.putAtt(nccid,Iiid,'miss_value',single(-9999));

netcdf.endDef(nccid);

nn=0;

ncid=netcdf.open([woddir,'ocldb1522513235.25662.CTD.nc'],'nowrite');

vid=netcdf.inqVarID(ncid,'cast');

cnm=netcdf.getVar(ncid,vid);

netcdf.close(ncid);

N=length(cnm); pto=0;

for j=1:length(cnm)

pt=round(j/N*1000)*0.1;

if pt>pto

disp([num2str(pt),'%']);

pto=pt;

end

snm=int2str(cnm(j));

for jj=length(snm):8

snm=cat(2,'0',snm);

end

flnm=['wod_',snm,'O.nc'];

ncid=netcdf.open([woddir,flnm],'nowrite');

[Ndim,Nvar]=netcdf.inq(ncid);

[~,Nprf]=netcdf.inqDim(ncid,0);

Cotry=' ';

Cruis=' ';

time=NaN;

date=NaN;

lat=NaN;

lon=NaN;

for k=0:Nvar-1

vnm=netcdf.inqVar(ncid,k);

if strcmp(vnm,'country')

Cotry=netcdf.getVar(ncid,k)';

Cotry=Cotry(1:13);

elseif strcmp(vnm,'WOD_cruise_identifier')

Cruis=netcdf.getVar(ncid,k)';

Cruis=Cruis(1:8);

elseif strcmp(vnm,'lat')

lat=netcdf.getVar(ncid,k);

elseif strcmp(vnm,'lon')

lon=netcdf.getVar(ncid,k);

elseif strcmp(vnm,'time')

time=netcdf.getVar(ncid,k);

elseif strcmp(vnm,'date')

date=netcdf.getVar(ncid,k);

elseif strcmp(vnm,'z')

z=netcdf.getVar(ncid,k);

elseif strcmp(vnm,'Temperature')

T=netcdf.getVar(ncid,k);

end

end

netcdf.close(ncid);

% Quality control (QC) is conducted to filter out any profile with the number of data points not greater than 10, the depth of the first data point (from the surface) greater than 80 meters, the depth of the last data point less than 20 meters, and the distance between the first and 10^th^ data points larger than 60 meters.

if length(z)>10 && z(1)<80 && z(end)>20 && z(10)-z(1)<60

iv=find(T>-5 & T<=36);

if length(iv)>8

[depp,tempp]=validata(z(iv),T(iv));

[ist,slop17,i17,dep,temp,isteps]=getgradient(depp,tempp);

if ~isempty(slop17)

[mld,Vmld]=ELGCore(dep,temp,ist,slop17,isteps,-1);

HC=Cp*refD*mld*Vmld;

if ~isnan(mld)

I=Iindex(dep,temp,mld,Vmld,length(i17));

end

netcdf.putVar(nccid,flid,[0,nn],[17,1],flnm');

netcdf.putVar(nccid,Cruisid,[0,nn],[8,1],Cruis');

netcdf.putVar(nccid,Cotryid,[0,nn],[13,1],Cotry');

netcdf.putVar(nccid,timeid,nn,1,time);

netcdf.putVar(nccid,timeid,nn,1,time);

netcdf.putVar(nccid,latid,nn,1,lat);

netcdf.putVar(nccid,lonid,nn,1,lon);

netcdf.putVar(nccid,TGrdid,nn,1,slop17);

netcdf.putVar(nccid,mxdid,nn,1,mld);

netcdf.putVar(nccid,MTid,nn,1,Vmld);

netcdf.putVar(nccid,HCid,nn,1,HC);

netcdf.putVar(nccid,Iiid,nn,1,I);

nn=nn+1;

end

end

end

end

netcdf.close(nccid);

***MATLAB function (validate.m) to filter out bad profiles***

function [dep,Var]=validata(dep,Var)

% function [dep,Var]=validata(dep,Var);

% filter out bad data

% Var: variable (potential density, temperature)

Var(dep<0)=[]; dep(dep<0)=[];

ii=find(diff(dep)<=0);

while(~isempty(ii))

dep(ii)=[]; Var(ii)=[];

ii=find(diff(dep)<=0);

end

ii=find(Var==inf);

if ~isempty(ii)

Var(ii)=[]; dep(ii)=[];

end

ii=find(abs(diff(Var)./diff(dep))>1);

if ~isempty(ii)

depp=dep(ii);

jj=find(depp>20,1);

if ~isempty(jj)

dep=dep(1:ii(jj)); Var=Var(1:ii(jj));

end

jj=find(depp<=20,1,'last');

if ~isempty(jj)

dep=dep(ii(jj)+1:end); Var=Var(ii(jj)+1:end);

end

end

***MATLAB function (getgradient.m) to calculate the vertical gradient between z_(0.1)_ and z_(0.7)_***

function [ist,slop17,i17,dep,Var,isteps,flag]=getgradient(dep,Var,TD)

% function [ist,slop17,i17,dep,Var,isteps,flag]=getgradient(dep,Var,TD);

% dep: depth (m) >0

% Var: variable (potential density, temperature)

% TD: 1; density, -1:temperature

% ist: starting index

% ied: ending index

% output:

% slop17: vertical gradient between z_(0.1)_ and z_(0.7)_

% i17: index between z_(0.1)_ and z_(0.7)_

% isteps: the steps used to calculate gradients

% flag: 0: normal

% 1: too few points (<=2) between 10 m and 40 m

% 2: too few total observation points (<=5)

% 3: maximum depth <20 m

% 4: starting observational point deeper than 50 m

% 5: temperature variance above 20 m > below 20 m

% 6: max difference < 1.0^o^C (temperature) or < 0.01 kg/m^3^ (potential density)

% 7: no thermocline

% 8: too small thermocline gradient

% 9: big temperature change between two neighboring vertical points (>5^o^C)

if ~exist('TD','var'), TD=-1; end

slop17=[]; i17=[]; isteps=[]; ist=[]; I30=[]; flag=0;

if max(abs(diff(Var)))>5

flag=9; return;

end

% Set up increment of the exponential steps: 2^n^ (n =1, …, 6)

Isteps=2.^(1:6);

ii=find(dep<=800); dep=dep(ii); Var=Var(ii);

ii=find(diff(dep)<=0);

while(~isempty(ii))

dep(ii)=[]; Var(ii)=[];

ii=find(diff(dep)<=0);

end

Varr=Var*TD;

% Return if the data are not good.

if length(dep)<6, flag=2; return; end

if dep(end)<20, flag=3; return; end

if dep(1)>50, flag=4; return; end

max20=max(Varr(dep<20))-min(Varr(dep<20));

maxdeep=max(Varr(dep>20))-min(Varr(dep>20));

if max20>maxdeep, flag=5; return; end

% Determine the starting depth z_1_ with the min gradient in upper 20 m.

ii=find(dep<20);

if(length(ii)>2)

slp=abs(diff(Varr(ii))./diff(dep(ii)));

[~,ist]=min(slp);

else

ist=1;

end

Vist=Varr(ist);

Varr=Varr-Vist;

[Vmax,imax]=max(Varr(ist:end));

imax=imax-1+ist;

% Estimate the number of vertical points in the thermocline.

if((Vist<40 && Vmax<=1) || (Vist>40 && Vmax<=0.01)), ist=[]; flag=6; return; end

i17=(find(Varr(ist:imax)<0.1*Vmax,1,'last'):find(Varr(ist:imax)>=0.7*Vmax,1))+ist-1;

n17=length(i17);

if(n17<4), ist=[]; flag=7; return; end

vv=Varr(i17(2:end))-Varr(i17(1));

dd=dep(i17(2:end))-dep(i17(1));

slps=vv./dd;

slop17=prctile(slps,50);

i17=i17(1)+(0:find(slps>=slop17,1,'last'));

n17=length(i17);

isteps=Isteps(Isteps<=n17);

if isteps(end)<32 && isteps(end)~=n17

isteps=cat(2,isteps,n17);

end

if((Vist<40 && slop17<1e-3) || (Vist>=40 && slop17<1e-5)), ist=[]; flag=8; return; end

% Update ist

vv=Varr(1:i17(1));

dv=abs(vv-mode(vv)); dv=dv(end:-1:1);

[~,ist]=min(dv); ist=length(dv)+1-ist;

if sum(dep>=10 & dep<=40)<2, flag=1;

end

***MATLAB function (ELGCore.m) to determine G and ITL depth***

function [mld,Vmld,imld]=ELGCore(dep,Var,ist,refslop,isteps,TD)

% function [mld,Vmld,imld]=ELGCore(dep,Var,ist,refslop,isteps,TD);

% input:

% dep: depth (m) >0

% Var: variable (potential density, temperature)

% ist: starting index

% ied: ending index

% isteps: exponential steps

% TD: temp:-1, density: +1

% output:

% mld: mixed layer depth (m) for density,

% mld: isothermal layer depth (m) for temperature

% Vmld: value at the mixed layer depth

% imld: I-index for the isothermal (mixed) layer depth

mld=NaN; Vmld=NaN; imld=NaN;

Var=Var*TD;

if isempty(ist), return; end

N=length(dep)-isteps(end);

slopmin=zeros(N,1);

for i=ist:N

dps=dep(i+isteps)-dep(i);

ii=find(dps>=2);

if length(ii)<3

slops=(Var(i+isteps)-Var(i))./dps;

else

Vs=Var(i+isteps(ii))-Var(i);

slops=Vs./dps(ii);

end

slopmin(i)=min(slops);

if slopmin(i)>0.8*refslop

mld=dep(i); Vmld=Var(i)*TD;

break;

end

end

if i==N

[~,imld]=max(slopmin);

mld=dep(imld); Vmld=Var(imld)*TD;

else

imld=i;

end

***MATLAB function (Iindex.m) to calculate the I_ITL_ for the technical validation***

function Ii=Iindex(dep,Var,mld,Vmld,n17)

% function Ii=Iindex(dep,Var,mld,Vmld,n17);

% input:

% dep: depth (m)

% Var: variable (potential density, temperature)

% mld: mixed layer depth (potential density)

% mld: isothermal layer depth (temperature)

% Vmld: mixed layer variable

% n17: vertical data points between z_1_ and z_(0.7)_

% output:

% Ii: Identification Index

if isnan(Vmld), Ii=NaN; return; end

if size(dep,1)==1

dep=dep'; Var=Var';

end

dep1=dep(dep<mld-0.001); dep2=dep(dep>mld+0.001);

V1=Var(dep<mld-0.001); V2=Var(dep>mld+0.001);

dep=cat(1,dep1,mld,dep2);

Var=cat(1,V1,Vmld,V2);

imld=find(abs(dep-mld)<0.0001);

i3=find(dep<=1.5*mld);

i3=i3(i3<=imld+n17);

if length(i3)<7

Ii=0; return;

end

i1=(1:imld)';

A1=0; A2=0;

if(imld>=3)

p1=polyfit(dep(i1),Var(i1),1);

v1=polyval(p1,dep(i1));

A1=sum((Var(i1)-v1).^2);

end

i2=i3(i3>imld);

if length(i2)>=3

p2=polyfit(dep(i2),Var(i2),1);

v2=polyval(p2,dep(i2));

A2=sum((Var(i2)-v2).^2);

end

p3=polyfit(dep(i3),Var(i3),1);

v3=polyval(p3,dep(i3));

A3=sum((Var(i3)-v3).^2)+eps;

Ii=max(0,1-(A1+A2)./A3);
